# Autonomic Modulation in Parkinson’s Disease Using Whole-Body Cryostimulation: A Pilot Study

**DOI:** 10.3390/biomedicines12112467

**Published:** 2024-10-27

**Authors:** Paolo Piterà, Riccardo Cremascoli, Laura Bianchi, Francesca Borghesi, Federica Verme, Stefania Cattaldo, Elisa Prina, Stefania Mai, Pietro Cipresso, Federica Galli, Jacopo Maria Fontana, Lorenzo Priano, Alessandro Mauro, Paolo Capodaglio

**Affiliations:** 1 Department of Neurosciences “Rita Levi Montalcini”, University of Turin, 10126 Turin, Italy; p.pitera@auxologico.it (P.P.); e.prina@auxologico.it (E.P.); lorenzo.priano@unito.it (L.P.); alessandro.mauro@unito.it (A.M.); 2 Laboratory of Clinical Neurobiology, IRCCS Istituto Auxologico Italiano, San Giuseppe Hospital, 28824 Verbania, Italy; r.cremascoli@auxologico.it (R.C.); laurabianchitnfp@gmail.com (L.B.); s.cattaldo@auxologico.it (S.C.); gallif.tnfp@gmail.com (F.G.); 3 Department of Psychology, University of Turin, 10124 Turin, Italy; f.borghesi@auxologico.it (F.B.); pietro.cipresso@unito.it (P.C.); 4 Research Laboratory in Biomechanics, Rehabilitation and Ergonomics, IRCCS Istituto Auxologico Italiano, San Giuseppe Hospital, 28824 Verbania, Italy; f.verme@auxologico.it (F.V.); j.fontana@auxologico.it (J.M.F.); 5 Laboratory of Metabolic Research, IRCCS Istituto Auxologico Italiano, San Giuseppe Hospital, 28824 Verbania, Italy; s.mai@auxologico.it; 6 Department of Surgical Sciences, Physical and Rehabilitation Medicine, University of Torino, 10126 Torino, Italy

**Keywords:** whole-body cryostimulation, parkinson’s disease, rehabilitation, heart rate variability, autonomic modulation

## Abstract

**Background:** Parkinson’s disease (PD) is a multifaceted neurodegenerative disorder that progressively affects both the central and peripheral nervous systems. This pilot study aimed to examine the effects of repeated whole-body cryostimulation (WBC) sessions on the sympathovagal balance in PD patients and correlate heart rate variability (HRV) indexes with peripheral biomarkers of the autonomic nervous system (ANS). **Methods:** Seventeen PD patients with mild to moderate motor severity underwent a 10-session WBC cycle over 5 consecutive days. Thirteen patients (6 males, 7 females; mean age 64.5 ± 9.01 years; mean disease duration 5.4 ± 2.3 years) completed the protocol. Cardiac autonomic activity was assessed through HRV measures including RR interval variability (RR mean, RR min, RR max), power density of high and low frequencies (HF, LF), RMSSD, and the LF/HF ratio. Systemic sympathetic activity was evaluated via circulating blood catecholamine levels. **Results**: Significant increases were observed in RR mean, RR min, RR max, RMSSD, and HF spectrum, indicating enhanced parasympathetic activity. Blood pressure remained stable, suggesting safety. **Conclusions:** These findings provide initial support to WBC as a potential “rehabilitation booster” in PD, enhancing sympathovagal balance. Further research is needed to explore the long-term benefits of WBC in PD management.

## 1. Introduction

Parkinson’s disease is one of the most common neurodegenerative disorders with a 200 year-long research history, characterized by early prominent death of dopaminergic neurons in the substantia nigra pars compacta [1]. The resultant dopamine deficiency within the basal ganglia leads to movement disorders characterized by classical parkinsonian motor symptoms [2]. Additionally, as a multisystem disorder, Parkinson’s disease is often associated with numerous non-motor symptoms, some of which can precede the motor dysfunction by more than a decade [3].

An increasing number of studies have recently focused on the role of autonomic dysfunction in the early diagnosis of PD, making this one of the top research frontiers in the PD field [4,5]. Autonomic dysfunction in PD includes gastrointestinal malfunction, urinary disturbance, sexual dysfunction, thermoregulatory aberrance, pupillo-motor and tear abnormalities, hypersalivation, swallowing difficulties, delayed gastric emptying, constipation and orthostatic hypotension [4].

In addition, patients with PD frequently exhibit cardiac autonomic abnormalities, which are associated with an increased risk of cardiovascular disease and respiratory failure—representing the two leading causes of mortality in this patient cohort [3,6].

In idiopathic Parkinson’s disease (PD), autonomic dysfunction is primarily associated with peripheral degeneration of postganglionic sympathetic fibers, although central autonomic structures, such as the brainstem, may also be involved in later stages [7]. Dysautonomia is also a significant feature in atypical parkinsonism, such as progressive supranuclear palsy (PSP) and multiple systemic atrophy (MSA). In MSA, severe autonomic dysfunction, particularly involving cardiovascular regulation, is a hallmark and includes profound cardiovascular dysregulation, such as orthostatic hypotension, whereas in PSP, more subtle autonomic features including urinary and gastrointestinal problems, have been documented [7,8]. Mechanistically, autonomic dysautonomia in MSA involves both central and peripheral autonomic pathways, whereas PSP primarily affects central structures such as the brainstem. Understanding these distinct and overlapping pathways may improve differential diagnosis and could lead to more targeted therapeutic approaches for the management of dysautonomia in PD and parkinsonian syndromes.

Assessing parasympathetic cardiovascular regulation in PD is a promising way to assess symptoms and to improve our knowledge of the course of the disease. Thus, testing the autonomic nervous system (ANS) in patients with PD is crucial for characterizing subgroups (with or without dysautonomia) and thereby forecasting the prognosis over time.

Heart rate variability (HRV) analysis is a simple method to estimate the overall tone of the autonomic nervous system [9]. Whereas heart rate quantifies the number of heartbeats per minute, HRV refers to the fluctuation in the time between successive heartbeats and it reflects the sympathetic and parasympathetic modulation of cardiac activity [10].

According to a recent literature review, PD patients show a strong decrease in parasympathetically modulated HRV parameters compared to healthy controls [9]. PD is usually associated with lower values of HF ms^2^ and lower values of RMSSD during short-term measurements [11]. Furthermore, there is some evidence that more advanced PD leads to an increasing reduction in these parasympathetically modulated HRV parameters [12]. In fact, patients with PD exhibit a progressive imbalance of the autonomic system, resulting in a predominant influence of either the sympathetic or the parasympathetic system [9].

Although autonomic dysfunction is one of the most common non-motor phenotypes in PD, it is very challenging to manage [13]. Current treatments for PD include medicinal treatment using levodopa and surgical treatment using deep brain stimulation. Although these treatments offer relief of symptoms, they do not cure the disease [14]. The limited treatment options available for autonomic dysfunction in PD make it one of the key issues in PD management [4].

Whole body Cryostimulation (WBC) is a physical medical treatment that consists of exposing the entire body to extremely low temperatures (ranging from −110 to −140 °C) in short sessions (lasting 2–3 min) in order to achieve several health benefits [15]. Very known and largely used in the sport realm, WBC is now emerging also in the clinical field, but it has never been tested on patients with PD. WBC has been indicated as a “training method” for the autonomic nervous system [16]. In fact, cold stimulus activates afferent signals from the peripheral receptors converging in the medial preoptic region of the hypothalamus, from which efferent signals cause reflex cutaneous vasoconstriction, leading to a shift in blood volume toward the core resulting in increased central pressure [16,17].

This effect is responsible for reducing sympathetic nerve activity through baroreflex activation and shifting autonomic control of heart rate toward parasympathetic dominance [18]. Remote from cold stimulation, an increase in parasympathetic cardiac control occurs even overnight [19]. Furthermore, in healthy subjects and in patients with other neurological disorders than PD, WBC proved to have beneficial effects on sleep quality, restless leg syndrome, and cognitive performance [20,21,22].

Since WBC has been shown to activate the parasympathetic component of the autonomic nervous system in healthy individuals via HRV testing [18,23,24,25], and given that PD leads to an increasing reduction in parasympathetically modulated HRV parameters, with cardiovascular dysautonomia indicated as a major cause of mortality in PD patients, we hypothesized that WBC could be evaluated as an adjunctive therapeutic tool in Parkinson’s disease rehabilitation.

The main aim of this study was to evaluate the effects of repeated WBC sessions on the sympathovagal balance in PD as assessed by HRV Test. A secondary aim was to correlate HRV indices with plasmatic catecholamines as peripheral biomarkers of the ANS. Since this is a pilot study, it also aims to provide preliminary data that can serve as a basis for future protocols with larger sample sizes. These preliminary results will help validate the observed effects and guide optimization of therapeutic strategies in subsequent, more comprehensive studies.

## 2. Materials and Methods

### 2.1. Participants

Between June 2021 and March 2024, adult inpatients with Parkinson’s disease admitted to the Neurologic Unit of San Giuseppe Hospital, IRCCS Istituto Auxologico Italiano, Piancavallo (VB), agreed to participate in this study. Patients were given full information about the scope and methodology of the study, which was conducted in conformity with the Declaration of Helsinki of the World Medical Association and approved by the Ethics Committee of the Istituto Auxologico Italiano (reference: 2021_05_18_14). Written and verbal informed consent was obtained from all patients.

The sample analyzed in this study represents a subset of a larger cohort originally collected to investigate broader trends in the impact of WBC in patients with metabolic or neurological disease or fibromyalgia or healthy normal weight/overweight patients (study registration: NCT05443100). The subgroup selected for this specific analysis was chosen based on the presence of a diagnosis of Parkinson’s disease.

### 2.2. Study Design

**Figure 1:**
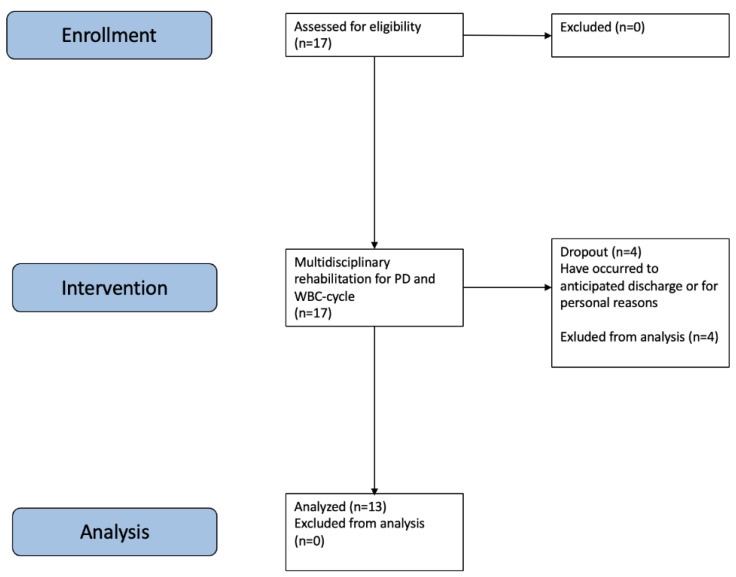
CONSORT flow chart describes the different stages of the study: patients’ enrollment, intervention, and data analysis.

This study aimed to investigate the effects of WBC on the ANS in PD patients. The stages of the protocol are described in Figure 1 and include patients’ enrollment, intervention, and data analysis. We consecutively recruited patients for a comprehensive, multidisciplinary rehabilitation program for PD, which included 10 WBC sessions in a medical nitrogen-cooling cryochamber (Arctic, CryoScience, Rome, Italy). Before starting the experiment, all the participants underwent a one-minute familiarization WBC treatment (T0) at −110 °C. Then, they underwent 10 WBC sessions, each lasting two minutes, over a 1-week period (2 treatments per day, Monday through Friday, at 8:15 a.m. before exercise classes and physical therapy, and at 12:00 p.m., before lunch). Before (PRE) and after (POST) the first (T1) and the last (T10) WBC session, all participants underwent Heart Rate Variability Test (as depicted in Section 2.6). To ensure the maximum accuracy of the results, patients were prevented from smoking cigarettes or consuming stimulating beverages such as coffee or tea on the morning of the HRV Tests (T1 and T10).

Additionally, blood pressure and blood samples were collected before and after these sessions. The Unified Parkinson’s Disease Rating Scale (UPDRS) Part III was administered to evaluate disease progression over time. Assessments were consistently conducted by a neurologist (R.C.) at the same time of day, specifically during the motor ON phase, which occurs when patients experience the full effect of their medication (taken at least 30 min prior to testing).

### 2.3. Participant Eligibility

Inclusion criteria for the study were as follows: a diagnosis of Parkinson’s disease without motor fluctuations (defined as the absence of motor OFF phases) or dementia (MMSE score ≥ 24); the ability to stand autonomously for at least 3 min, a stable daily dose of levodopa with no changes during the cryotherapy treatment cycle.

The levodopa daily dose ranged between 300 and 1250 mg of levodopa equivalent daily dose (LEDD), which calculates the contribution made by all antiparkinsonian drugs (levodopa and dopamine agonists).

Only patients with idiopathic Parkinson’s disease were recruited, particularly those with the bradykinetic-rigid subtype, and with a range 1–3 according to the Hoehn and Yahr Scale for disease severity.

Exclusion criteria included severe psychiatric conditions, active neoplasia, or a recent history of malignancy, acute respiratory or cardiovascular disease, unstable hypertension, cold intolerance, asthma, claustrophobia, recent modification of usual drug treatment, cryoglobulinemia, previous treatment with WBC, undesired weight loss in the last 3 months, and a body temperature greater than 37.5 °C.

Furthermore, a specific screening was to exclude patients who were taking monoamine oxidase inhibitors (MAOIs), and antipsychotic drugs. 

Patients with atypical parkinsonism, such as multiple systemic atrophy (MSA) or progressive supranuclear palsy (PSP), were excluded to ensure homogeneity of the sample and avoid confounding effects from other forms of parkinsonism. 

Prior to the experiment, a physician (R.C.) examined all participants to ensure they met the inclusion criteria and had no contraindications to WBC. Only those who were approved by the physician were enrolled in the research study.

### 2.4. Multidisciplinary Rehabilitation Intervention

The comprehensive rehabilitation program (average duration of 7 days) encompassed personalized nutritional intervention, psychological assistance, a 10-session WBC cycle and closely monitored physical activities during patients’ hospitalization. Following an initial dietary assessment, patients were prescribed a personalized, balanced, Mediterranean diet ranging from 1300 to 1500 kcal. This diet consisted of: Proteins: 70–74 g (20–21% of total daily kcal); Fats: 42–47 g (29–30% of total daily kcal, with less than 8% saturated fat); Carbohydrates: 162–190 g (50–51% of total daily kcal, with less than 15% from simple sugars); Fiber: 30 g primarily sourced from fresh vegetables. Furthermore, each patient underwent a personalized physiotherapy program tailored to their individual needs, disease stage, and capabilities. Daily 60-min physiotherapy sessions included progressive aerobic training, postural and balance control exercises, and strengthening exercises. All exercises were performed under the supervision of a physiotherapist and were suspended if a score of 5 on the Borg scale was reached. This approach was individualized based on the patient’s physical fitness, clinical status, and subjective perception of fatigue.

### 2.5. Description of the WBC Session

WBC session consisted of exposing the entire body to cryogenic temperatures at −110 °C for 2 min in a medical liquid nitrogen cooling cryochamber (Artic, CryoScience, Rome, Italy) located at IRCCS Istituto Auxologico Italiano, in Piancavallo, Italy. The familiarization treatment consisted of 1 min at −110 °C. Subjects were asked to remove glasses, contact lenses, and jewelry and were instructed to dry their bodies thoroughly to avoid risk of skin cold-lesions. Patients entered the cryochamber minimally dressed, wearing shorts or sweatpants, light T-shirt or shirtless (sports bras for women). Body extremities were protected with mid-calf socks, clogs, gloves, headgear, and earmuffs. To protect the oral and nasal mucosa from the extreme cold temperatures and for hygiene reasons, the patients were requested to wear a surgical mask during WBC treatment. Subjects were instructed to breathe normally in the cryochamber and, if needed, to shift their weight and move their fingers to better tolerate WBC treatment. Visual and vocal contact with the patients was maintained during all the WBC sessions duration. For safety reasons, systolic and diastolic blood pressure were measured before and after each treatment. Each WBC session was performed and controlled by specifically trained personnel (P.P.; F.V).

### 2.6. Heart Rate Variability Test and Blood Pressure

Blood pressure, heart rate, and HRV were recorded before and after the first WBC session (PRE-POST T1) and before and after the last WBC session (PRE-POST T10). For each measurement condition, participants were comfortably positioned in a supine position on a medical bed for 15 min. This test was conducted in a quiet room, to avoid HR fluctuations. Additionally, the participants were asked to remain still and not to talk. Lab Chart software, version 6Pro (ADInstruments, Bella Vista, NSW, Australia) was used for data analysis using the HRV module. An experienced investigator (L.B.) visually identified and manually removed any occasional ectopic beats and artifacts. R waves were sensed by an individually adjusted threshold. HRV was analyzed off-line on the software with HRV module. Time-domain parameters were determined by R-R inter-beat intervals and included: mean heart rate (mean HR); mean of the normal to normal heartbeats (mean RR) that reflects an estimate of parasympathetic regulation of the heart; standard deviation of the normal to normal heartbeats (SDNN) that measures overall heart rate variability; and the square root of the mean squared differences of successive RR intervals (RMSSD), which reflects parasympathetic modulation of the autonomic system. HRV in the high-frequency range is so rapid that it may only be mediated by the parasympathetic nervous system. However, part of the high-frequency power seems to be caused by respiration-induced changes in intrathoracic pressure and blood volume. In the low-frequency range, both the sympathetic and the parasympathetic nervous systems can affect HRV. Our analysis was, therefore, restricted to HRV indicators of parasympathetic modulation, namely the power density of high frequency (HF), the root-mean square difference of successive normal R–R intervals (RMSSD), a time-varying index, and the LF/HF ratio. Mean HR was also analyzed.

The HRV test consisted of three phases:

1. Resting Condition: The patient laid supine for 10 min, relaxed with spontaneous breathing.
2. Deep Breathing Test: The patient remained supine for 3 min with controlled deep breathing. Participants were instructed to breathe according to the slow breathing technique during the measurements. Slow breathing is performed at a slower pace (around 6 cycles per minute, cpm) than spontaneous breathing, which usually ranges from 12 to 20 cpm in adults, and typically with a higher breathing amplitude [26].
3. Lying to Standing: Initial blood pressure was measured while the patient was lying down. The patient was then instructed to move from a supine to a standing position as quickly as possible, maintaining this position for a period of five minutes. Blood pressure was then re-measured one minute after the patient had assumed the standing position, and a third measurement was taken at the conclusion of the five-minute period.

During each of the three ECG recording phases, the following parameters were analyzed:

- RR max interval
- RR min interval
- RR mean interval

In the first phase, a complete HRV study was conducted, including measurements of SDNN, RMSSD, HF, LF, HF/LF ratio, and Total Spectrum Frequency [10]. In the Deep Breathing phase, the RR max, min, and mean intervals were used to calculate the RR Interval Variability Index (RRIV = (RR max − RR min) × 100/RR mean). In the Lying-to-Standing phase, the ratio of RR max to RR min was calculated. 

### 2.7. Blood Sample Collection

Blood samples were collected in the morning in fasting condition, just before and after the first WBC session (within 15 min) and repeated before and after the last WBC treatment in the same conditions. Fasting blood samples were collected from a superficial forearm vein using standard venipuncture technique. For each blood sampling, ~3.5 mL was directly collected into EDTA tubes. Blood samples were immediately centrifuged (10 min, 4200 rpm at 4 °C), and the obtained EDTA-plasma samples were then stored in multiple aliquots (500–1200 μL per samples) at − 80 °C until future analysis. From these samples, epinephrine, norepinephrine, and dopamine were determined in plasma by enzyme-linked immunosorbent assay with commercially available high sensitivity ELISA kits (TRICAT, Tecan-IBL INTERNATIONAL-Hamburg, Germany). All blood samples were analysed in duplicate at specific wavelength (450 nm) on a spectrophotometer ( iMark Microplate reader; BIO-RAD Laboratories, Inc. USA). Intra-assay coefficients of variation were 6.8%, 7.4%, 10.9% for epinephrine, norepinephrine, and dopamine, respectively. Analytical sensitivity was 8, 20, and 4 pg/mL for epinephrine, norepinephrine, and dopamine, respectively. All the analyses were performed according to the instructions available with the kit. 

### 2.8. Feasibility

Compliance with WBC protocol was closely monitored, and test and questionnaire completion rates were recorded before and after the intervention. Adverse events were monitored throughout the duration of the trial, and we did not register any. The dropouts occurred due to personal reasons and not because of poor tolerance to the rehabilitation protocol or the WBC cycle, which, instead, were very well tolerated by all the subjects participating in this study.

### 2.9. Study Variables

The primary outcomes considered for this study were the Heart Rate Variability (HRV) Test variables, which included RR mean, RR min, RR max, RRIV, RR max/min stand up, total spectrum, High Frequency (HF) spectrum, Low Frequency (LF) spectrum, LF/HF ratio, SDNN, and RMSSD. 

Secondary outcomes included plasmatic catecholamines, blood pressure measurement, and the Unified Parkinson’s Disease Rating Scale (UPDRS) part III.

**Figure 2:**
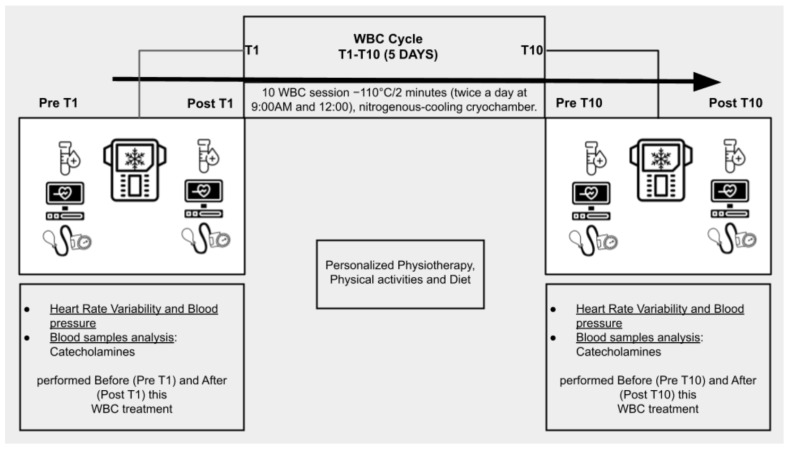
WBC rehabilitation protocol.

The detailed diagram of the protocol can be found in Figure 2.

### 2.10. Statistical Analysis

A full within repeated measures analysis of variance (ANOVA) was conducted to evaluate the effects of Long run (T1 vs. T10) and Short run (Pre vs. Post) on various cardiovascular and biochemical parameters. Post-hoc analysis used Tukey correction. Jamovi 2.3.21 was used as statistical software. A paired *t*-test was used to evaluate the difference between UPDRS T1 vs. T10. 

## 3. Results

### 3.1. Demographic and Baseline Characteristics

A total of 17 adult inpatients (8 females, 9 males) with Parkinson’s disease agreed to participate in this study. Thirteen patients (7 females, 6 males), with a mean age of 64.5 ± 9.01 years, completed the entire research protocol and were included in the analysis. Table S1 details the baseline characteristics of the participants.

### 3.2. Heart Rate Variability Test Variables

#### 3.2.1. Time-Domain Indices

ANOVA tests revealed significant interactions and main effects for several parameters.

For RR mean (ms), there was a significant effect of Short run (Pre vs. Post) (F (1, 12) = 41.82, *p* < 0.001, η^2^p = 0.77). Post hoc comparisons, with Tukey correction, revealed significant differences between Pre-T1 and Post-T1 (t (12) = −5.59, *p* < 0.001); Pre-T1 vs. Post-T10 (t (12) = −4.47, *p* = 0.004); Pre-T10 vs. Post-T10 (t (12) = −6.07, *p* < 0.001). Table 1 shows the RR mean descriptives. 

For RR Min (ms), there was a significant effect of Short run (Pre vs. Post) (F (1, 12) = 8.49, *p* = 0.0013, η^2^p = 0.414). No post hoc comparisons, with Tukey correction, were significant. Table 2 shows the RR min descriptives. 

For RR Max (ms), there was a significant effect of Short run (Pre vs. Post) (F (1, 12) = 15.27, *p* = 0.002, η^2^p = 0.560. Post hoc comparison, with Tukey correction, shows a significant effect for T1 Pre vs. T10 post (t (12) = −2.989, *p* = 0.048); T1 Post vs. T10 Post (t (12) = −3.265, *p* = 0.030); T10 Pre vs. T10 Post (t (12) = −6.099, *p* < 0.001). Table 3 shows the RR max descriptives. 

For RMSSD (ms), there was a significant effect of Short run (Pre vs. Post) (F (1, 12) = 10.50, *p* = 0.007, η^2^p = 0.467). Post hoc comparisons, with Tukey correction, revealed significant differences between T1 Pre and T10 Post (t (12) = −3.026, *p* = 0.045). Table 4 shows the RMSSD descriptives. 

For RRIV and RR max/RR min stand up we observed no significant difference. 

**Figure 3:**
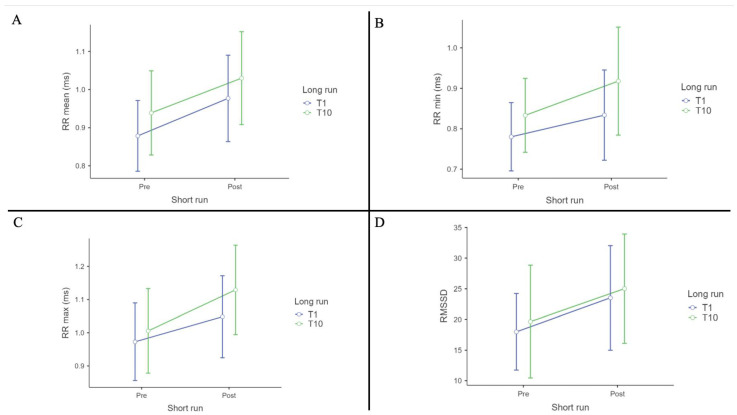
(**A**): RR mean (ms); (**B**): RR min (ms), (**C**): RR max (ms) and (**D**): RMSSD (ms) ANOVA results.

RR mean, RR max, RR min and RMSSD ANOVA results are depicted in Figure 3.

#### 3.2.2. Frequency-Domain Indices

For HF Spectrum (ms^2^), there was a significant main effect of Short run (Pre vs. Post) (F (1, 12) = 11.44, *p* = 0.005, η^2^p = 0.488). Post hoc comparisons, with Tukey correction, revealed significant differences between T1 Pre and T10 Post (t (12) = −3.120, *p* = 0.039). Table 5 shows the HF spectrum descriptive. 

For LF Spectrum (ms^2^), there was a significant effect of Short run (Pre vs. Post) (F (1, 12) = 4.456, *p* = 0.056, η^2^p = 0.271). No post hoc comparisons were significant. Table 6 shows LF spectrum descriptives.

For Total Spectrum Frequency (ms^2^), there was a marginally significant effect of Long run (F (1, 12) = 4.43, *p* = 0.057, η^2^p = 0.270). The Short run (Pre vs. Post) had a significant effect (F (1, 12) = 5.36, *p* = 0.039, η^2^p = 0.309). Post hoc comparisons, with Tukey correction, revealed significant differences between T1 Pre and T10 Post (t (12) = −3.417, *p* = 0.023). Table 7 shows the Total spectrum Frequency descriptives.

**Figure 4:**
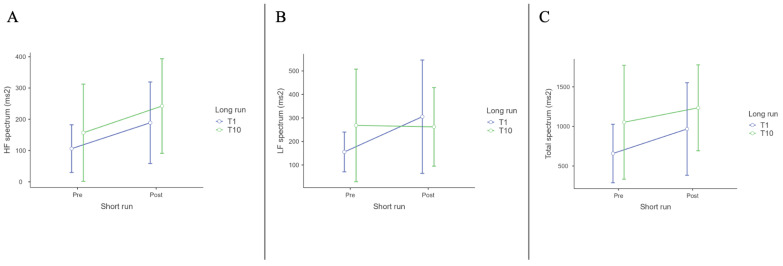
(**A**): HF (ms^2^); (**B**): LF (ms^2^); (**C**): total spectrum (ms^2^) ANOVA results.

HF spectrum, LF spectrum and Total spectrum frequencies results are shown in Figure 4.

### 3.3. Blood Pressure

Repeated ANOVA results indicate that there were no significant effects of Long run (T1 vs. T10) and Short run (Pre vs. Post) for both systolic and diastolic blood pressure in either the supine position or after 5 min of standing (*p*-values > 0.05). Blood pressure (mmHg) results are depicted in Table 8.

### 3.4. Plasmatic Catecholamines

**Figure 5:**
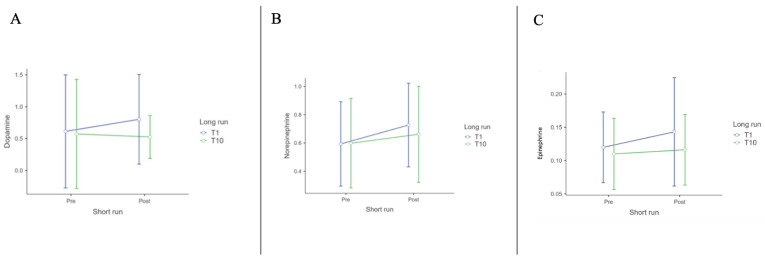
(**A**): Dopamine (ng/mL); (**B**): Norepinephrine (ng/mL) and (**C**): Epinephrine (ng/mL) ANOVA results.

No significant variation was found for catecholamines. However, we observed an increasing trend from Pre-T1 to Post-T1 and a decreasing trend by the end of the protocol, with Post-T10 levels returning to values similar to Pre-T1. Catecholamine descriptives are presented in Table 9; Figure 5 shows the trend of Dopamine, Norepinephrine, and Epinephrine over sessions.

### 3.5. UPDRS

A paired sample t-test showed a non-significant effect of Time (Pre T1 vs. Post T10) on Disease rating, assessed with part III UPDRS (*p* = 0.685). Results of UPDRS are presented in Figure S1 and the descriptives are presented in Table S2.

## 4. Discussion

The present study investigated the effects of Whole Body Cryostimulation on sympathovagal balance in patients with Parkinson’s disease. To the best of our knowledge, this is the very first study addressing this issue. 

The significant changes in heart rate variability (HRV) parameters observed in this study suggest that WBC might help improve cardiac autonomic modulation in PD patients. Specifically, significant increases in RR mean, RR min, RR max, and RMSSD were observed, indicating enhanced parasympathetic activity. This finding aligns with previous research that demonstrated that cold exposure can shift autonomic control of heart rate toward parasympathetic dominance through baroreflex activation [18]. Louis et al. in fact, investigated the effects of repetitive exposure to WBC in healthy males with HRV test, finding a systematic decrease in HR (which corresponds to an increase in the RR interval) following each exposition and increased RMSSD over the treatments. Furthermore, they observed a rise in norepinephrine blood concentration after the first treatment returning to baseline level at the end of the protocol, suggesting a habituation effect after consecutives treatments. Similarly, our results in hematological catecholamines suggest that WBC might variably affect different aspects of neurochemistry in PD. Although there were no significant variations in noradrenaline, adrenaline, and dopamine levels, the trends observed—an increase from Pre-T0 to Post-T0 followed by a decrease by the end of the protocol—warrant further investigation. These trends might indicate an initial stress response to cold exposure, followed by physiological habituation, which aligns with the observed lower sympathetic response by the end of the WBC cycle and confirms the previous findings of Louis et al. [18]. The reduction in skin temperature following WBC triggers a series of physiological responses, ultimately increasing parasympathetic tone [18]. Cold-sensitive cutaneous thermoreceptors are stimulated, causing sympathetic activation and the release of catecholamines. This leads to peripheral vasoconstriction, redirecting blood flow to core organs, and raising blood pressure. Subsequently, compensatory mechanisms are activated to regulate blood pressure, resulting in vagally mediated bradycardia [18,27].

Our results demonstrated no significant changes in blood pressure, with no significant variations in systolic and diastolic blood pressure, both in supine and standing positions, suggesting that WBC is well-tolerated by PD patients and does not induce adverse cardiovascular effects. The absence of blood pressure fluctuations during and after WBC is particularly relevant for safety considerations, as approximately 50% of Parkinson’s disease patients experience autonomic insufficiency, including orthostatic hypotension [28]. Therefore, this treatment appears safe and does not exacerbate the cardiovascular instability often observed in this patient population. Maintaining stable blood pressure is in fact crucial in this context, as orthostatic hypotension can lead to dizziness, falls, and other complications [29].

The results regarding the Unified Parkinson’s Disease Rating Scale (UPDRS) part III did not show a significant effect of time, suggesting that the short-term WBC intervention did not substantially impact motor severity as measured by UPDRS. This might be also due to the short duration of the intervention and longer-term studies are necessary to establish whether WBC impacts on the motor component in PD patients.

Our findings support the hypothesis that WBC holds the potential to become a novel “rehabilitation booster” in PD, particularly concerning autonomic modulation [16]. By enhancing parasympathetic activity and maintaining stable blood pressure, WBC might contribute to improved cardiovascular health and potentially mitigate some non-motor symptoms associated with PD. However, further research with larger sample sizes and longer follow-up periods is needed to confirm these findings and unveil the long-term effects of WBC on autonomic function and motor experiences in daily life in PD. This study has several limitations that should be acknowledged. The small sample size and the absence of a control group limit the generalizability of the findings and the statistical power of the analysis. Another limitation of our study is the variability in patient age. Although age-related factors may potentially influence autonomic responses, our sample size does not allow us to fully explore this variable. As a pilot study, these limitations were expected; however, the results provide preliminary insights into the potential efficacy of WBC in Parkinson’s disease rehabilitation and require further studies to confirm these findings. Future studies with larger and more homogeneous age groups will be necessary to better understand the role of age in autonomic modulation with WBC.

In conclusion, this study provides initial innovative evidence regarding the positive impact of repeated WBC sessions on autonomic function in PD patients, as evidenced by significant improvements in HRV parameters. These findings provide initial evidence that needs to be confirmed by further investigations on WBC as a complementary therapeutic approach in the management of PD. Future studies should also aim to understand the mechanisms underlying these effects and assess the clinical relevance of WBC in improving functional outcomes for individuals with PD. Longer-term, controlled studies with larger sample size that include follow-up assessments will be needed to study the impact of WBC on short-term and long-term outcomes and to understand the potential therapeutic role of WBC in the management of PD.

## 5. Conclusions

The results of this pilot study show that a 10-session WBC cycle may help modulate cardiac autonomic function in patients with PD, confirming that repeated cold exposure can shift autonomic control of heart rate toward parasympathetic dominance also in patients with PD. This study also showed that WBC is a safe, well tolerated treatment that appears promising in managing autonomic dysfunction in PD patients.

## Figures and Tables

**Table 1:** RR mean descriptives (mean, median, SD, minimum, maximum; N = 13) measured before and after the first (T1) and last (T10) WBC sessions.

	N	Mean	Median	SD	Minimum	Maximum
RR mean Pre T1 (ms)	13	0.878	0.859	0.154	0.621	1.25
RR mean Post T1 (ms)	13	0.977	0.993	0.188	0.64	1.33
RR mean Pre T10 (ms)	13	0.939	0.953	0.183	0.72	1.31
RR mean Post T10 (ms)	13	1.03	1.018	0.202	0.759	1.39

**Table 2:** RR min descriptives (mean, median, SD, minimum, maximum; N = 13) measured before and after the first (T1) and last (T10) WBC sessions.

	N	Mean	Median	SD	Minimum	Maximum
RR min Pre T1 (ms)	13	0.78	0.79	0.14	0.604	1.13
RR min Post T1(ms)	13	0.834	0.817	0.185	0.503	1.14
RR min Pre T10 (ms)	13	0.833	0.803	0.151	0.653	1.2
RR min Post T10 (ms)	13	0.918	0.922	0.221	0.528	1.3

**Table 3:** RR max descriptives (mean, median, SD, minimum, maximum; N = 13) measured before and after the first (T1) and last (T10) WBC sessions.

	N	Mean	Median	SD	Minimum	Maximum
RR max Pre T1 (ms)	13	0.973	0.962	0.193	0.649	1.32
RR max Post T1 (ms)	13	1.048	1.05	0.204	0.661	1.44
RR max Pre T10 (ms)	13	1.006	0.999	0.211	0.761	1.44
RR max Post T10 (ms)	13	1.129	1.079	0.223	0.805	1.57

**Table 4:** RMSSD descriptives (mean, median, SD, minimum, maximum; N = 13) measured before and after the first (T1) and last (T10) WBC sessions.

	N	Mean	Median	SD	Minimum	Maximum
RMSSD Pre T1 (ms)	13	18	15.4	10.3	3.06	34.1
RMSSD Post T1 (ms)	13	23.5	25	14.1	3.42	56.3
RMSSD Pre T10 (ms)	13	19.6	17	15.2	6.97	63.8
RMSSD Post T10 (ms)	13	25	25.3	14.8	6.25	51.3

**Table 5:** HF spectrum descriptives (mean, median, SD, minimum, maximum; N = 13) measured before and after the first (T1) and last (T10) WBC sessions.

	N	Mean	Median	SD	Minimum	Maximum
HF spectrum (ms^2^) Pre T1	13	106	46	126	2.77	376
HF spectrum (ms^2^) Post T1	13	189	141	216	2.03	789
HF spectrum (ms^2^) Pre T10	13	157	67.5	257	10.03	975
HF spectrum (ms^2^) Post T10	13	242	148.3	251	13.89	716

**Table 6:** LF spectrum descriptives (mean, median, SD, minimum, maximum; N = 13) measured before and after the first (T1) and last (T10) WBC sessions.

	N	Mean	Median	SD	Minimum	Maximum
LF spectrum (ms^2^) Pre T1	13	155	105	140	2.62	486
LF spectrum (ms^2^) Post T1	13	305	173	399	3.49	1529
LF spectrum (ms^2^) Pre T10	13	268	136	396	45.96	1456
LF spectrum Post (ms^2^) T10	13	262	205	277	25.63	1047

**Table 7:** Total spectrum frequency descriptives (mean, median, SD, minimum, maximum; N = 13) measured before and after the first (T1) and last (T10) WBC sessions.

	N	Mean	Median	SD	Minimum	Maximum
Tot spectrum (ms^2^) Pre T1	13	658	466	612	67.3	2036
Tot spectrum (ms^2^) Post T1	13	968	702	968	36.2	3730
Tot spectrum (ms^2^) Pre T10	13	1053	511	1190	181.3	3854
Tot spectrum (ms^2^) Post T10	13	1235	1017	898	151	2780

**Table 8:** Blood pressure descriptives (mean, median, SD, minimum, maximum; N = 13) in supine and standing positions before and after the first (T1) and last (T10) WBC sessions.

Position	Variable	PRE T1 (Mean ± SD)	PRE T10 (Mean ± SD)	POST T1 (Mean ± SD)	POST T10 (Mean ± SD)
supine	SBP (mmHg)	119.2 ± 11.83	113.9 ± 13.12	115.5 ± 10.81	117.5 ± 11.86
supine	DBP (mmHg)	78.6 ± 6.58	76.8 ± 8.91	74.6 ± 17.83	77.9 ± 7.88
standing	SBP (mmHg)	116.1 ± 13.53	117.1 ± 11.87	114.3 ± 13.03	112.4 ± 12.16
standing	DBP (mmHg)	78.4 ± 9.38	80.6 ± 8.40	83.4 ± 6.53	78.1 ± 9.44

**Table 9:** Plasmatic Catecholamines descriptives (mean, median, SD, minimum, maximum; N = 12) measured before and after the first (T1) and last (T10) WBC sessions.

	N	Mean	Median	SD	Minimum	Maximum
NE pre T1 (ng/mL)	12	0.594	0.459	0.47	0.096	1.59
NE post T1 (ng/mL)	12	0.727	0.648	0.465	0.157	1.67
NE pre T10 (ng/mL)	12	0.599	0.389	0.498	0.083	1.6
NE post T10 (ng/mL)	12	0.662	0.568	0.535	0.121	1.92
E pre T1 (ng/mL)	12	0.12	0.0935	0.0836	0.017	0.294
E post T1 (ng/mL)	12	0.143	0.0835	0.1281	0.022	0.41
E pre T10 (ng/mL)	12	0.11	0.103	0.0843	0.035	0.324
E post T10 (ng/mL)	12	0.116	0.1025	0.0834	0.012	0.31
Dopa pre T1 (ng/mL)	12	0.738	0.243	1.332	0.053	4.57
Dopa post T1 (ng/mL)	12	0.785	0.475	1	0.03	3.66
Dopa pre T10 (ng/mL)	12	0.605	0.233	1.219	0.073	4.4
Dopa post T10 (ng/mL)	11	0.525	0.409	0.501	0.07	1.84

Abbreviation list: NE: norepinephrine (ng/mL); E: epinephrine (ng/mL); Dopa: dopamine (ng/mL).

## Data Availability

Data are available on request in Zenodo repository https://doi.org/10.5281/zenodo.13772217 (accessed on 27/10/2024) due to restrictions, e.g., privacy or ethical.

## Supplementary Materials

The following supporting information can be downloaded at: https://www.mdpi.com/article/10.3390/biomedicines12112467/s1, Table S1: Baseline characteristics of participants composing the experimental group; Table S2: UPDRS III descriptives (mean, median, SD, minimum, maximum; N = 8) measured before the first (T1) and after the last (T10) WBC sessions; Figure S1: UPDRS III results.
