# Supplementary material for: Autonomic Modulation in Parkinson’s Disease Using Whole-Body Cryostimulation: A Pilot Study
Source: Biomedicines. 2024 Oct 27;12(11):2467. doi: 10.3390/biomedicines12112467 (PMC11591671; doi:10.3390/biomedicines12112467)
Supplement: Supplementary file 1 [file biomedicines-12-02467-s001.zip › biomedicines-3268850-supplementary.pdf]

**Table S1**. Baseline characteristics of participants composing the experimental group (mean, median, SD, minimum, maximum; N=13)

| | N | mean | median | SD | minimum | maximum |
|---|---|---|---|---|---|---|
| Age (years) | 13 | 64.54 | 63 | 9.01 | 47 | 83 |
| Mean disease duration (years) | 5 | 5.40 | 7 | 7.3 | 2 | 7 |
| Height (m) | 13 | 1.72 | 1.72 | 0.1 | 1.60 | 1.89 |
| Weight (Kg) | 13 | 75.8 | 71.5 | 10.16 | 61 | 93.8 |
| BMI (kg/m$^2$) | 13 | 26.18 | 25 | 3.91 | 21 | 35 |
| UPDRS (III) | 8 | 27.63 | 28.5 | 1 | 11 | 45 |
| RR mean (ms) | 13 | 0.878 | 0.859 | 0.154 | 0.621 | 1.25 |
| RR min (ms) | 13 | 0.780 | 0.790 | 0.140 | 0.604 | 1.13 |
| RR max (ms) | 13 | 0.973 | 0.962 | 0.193 | 0.649 | 1.32 |
| RMSSD (ms) | 13 | 17.982 | 15.377 | 10.340 | 3.065 | 34.08 |
| SDNN (ms) | 13 | 31.055 | 29.726 | 11.612 | 10.644 | 48.98 |
| HF spectrum (ms$^2$) | 13 | 106.102 | 46.011 | 126.325 | 2.772 | 375.50 |
| LF spectrum (ms$^2$) | 13 | 155.419 | 104.575 | 139.782 | 2.621 | 468.38 |
| Ratio LF/HF | 13 | 3.362 | 1.709 | 4.432 | 0.644 | 15.07 |
| supine SBP (mmHg) | 13 | 119.2 | 118 | 11.83 | 98 | 135 |
| standing SBP (5′) (mmHg) | 7 | 116.1 | 119 | 13.53 | 89 | 130 |
| supine DBP (mmHg) | 13 | 76.8 | 79 | 8.91 | 61 | 95 |
| standing DBP (5′) (mmHg) | 7 | 78.4 | 79 | 9.38 | 63 | 95 |
| Epinephrine (ng/mL) | 12 | 0.120 | 0.093 | 0.0083 | 0.017 | 0.294 |
| Norepinephrine (ng/mL) | 12 | 0.594 | 0.459 | 0.469 | 0.09 | 1.590 |
| Dopamine (ng/mL) | 12 | 0.738 | 0.243 | 1.331 | 0.053 | 4.570 |

**Table S1 abbreviation list**: BMI: Body Mass Index; DBP: Diastolic Blood Pressure; HF: High Frequency; LF: Low Frequency; LF/HF: Low Frequency and High Frequency Ratio; RMSSD (ms): Root Mean Square of the Successive Differences; RR: R-R Interval; SBP: Systolic Blood Pressure; SD: Standard Deviation; SDNN (ms): Standard Deviation of NN intervals; UPDRS (III): Unified Parkinson's Disease Rating Scale, Part III

**Table S2**. UPDRS III descriptives (mean, median, SD, minimum, maximum; N=8) measured before the first (T1) and after the last (T10) WBC sessions.

| | N | Mean | Median | SD | Minimum | Maximum |
|---|---|---|---|---|---|---|
| UPDRS Pre T1 | 8 | 27.6 | 28.5 | 10.5 | 11 | 45 |
| UPDRS Post T10 | 8 | 28 | 29 | 11.3 | 11 | 48 |

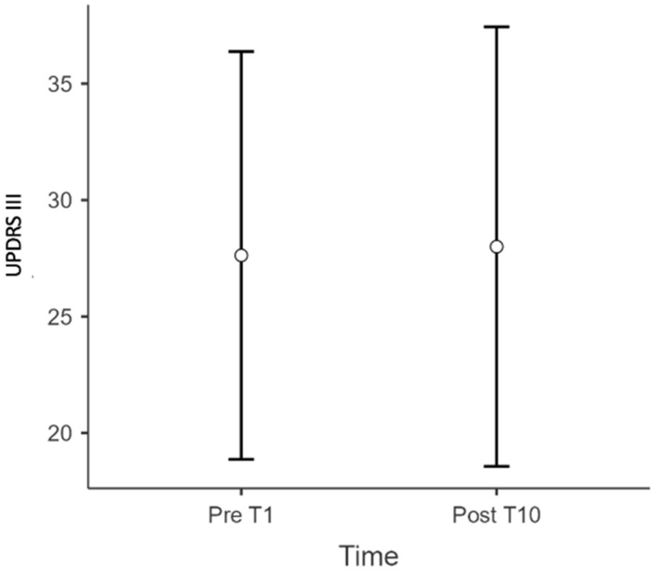


**Figure S1.** UPDRS III results
